# Prevalence of high and low risk HPV genotypes among vaccinated and non-vaccinated people in Tehran

**DOI:** 10.1186/s12985-023-02270-1

**Published:** 2024-01-05

**Authors:** Amir Gholamzad, Niloofar Khakpour, Mehrdad Hashemi, Mehrdad Gholamzad

**Affiliations:** 1 Farhikhtegan Medical Convergence Sciences Research Center, Farhikhtegan Hospital Tehran Medical Sciences, Islamic Azad University, Tehran, Iran; 2 Department of Laboratory Medicine, Faculty of Paramedical Sciences, Tehran Medical Sciences, Islamic Azad University, Tehran, Iran; 3 Department of Bacteriology and Virology, Shiraz University of Medical Sciences, Shiraz, Iran; 4 Department of Genetics, Faculty of Advanced Science and Technology, Tehran Medical Sciences, Islamic Azad University, Tehran, Iran; 5 Department of Microbiology and Immunology, Faculty of Medicine, Tehran Medical Sciences, Islamic Azad University, Tehran, Iran

**Keywords:** HPV, Genotyping, Prevalence, Gardasil, Cervical cancer, Vaccine, Vaccinations

## Abstract

**Background:**

Human Papillomavirus (HPV) is a prevalent STI (Sexually Transmitted Infection) that is estimated almost all sexually active Patients at some stage of their life will be infected by the virus. Although most HPV infections resolve spontaneously, some can result in health complications, such as genital warts and several types of cancer. This study analyzed the variety of HPV genotypes in females and males among the infected population.

**Methods:**

Samples were obtained from the oral, vaginal, and genital sites of study participants and the samples underwent DNA extraction and subsequently amplified using Real-Time PCR. The recognition of high-risk (HR) and low-risk (LR) HPV genotypes was carried out using the HPV REALQUALITY RQ-Multi diagnostic kit and demographic information was analyzed alongside statistical virological data.

**Results:**

Out of 936 samples, 324 cases (34.6%) were found to be positive for HPV, while 612 cases (65.4%) were negative. Of our participants, 70 samples of males (27.5%) and 254 samples of females (37.3%) were HPV-positive. Common genotypes included 16, 6, 11, and 18, while genotypes 59, 56, 31, 45, and 52 were also detected.

**Conclusion:**

According to the findings of this study, a significant prevalence of HPV infection was seen in males and females, and the incidence of high-risk genotypes was more diverse in males. While the vaccine was effective in preventing some types of HPV, such as 16, 18, 6, and 11, there seems to be an increase in infections caused by other genotypes, and precautions should be taken to prevent future health problems.

## Introduction

Human Papillomavirus (HPV) is a member of the *Papillomaviridae* family. This virus has a double-stranded DNA that can be divided into two groups: low-risk HPVs (LR HPVs) and high-risk HPVs (HR HPVs). LR-HPVs mainly cause cutaneous and anogenital warts, while HR-HPVs have been scientifically proven to be a factor in the occurrence of different forms of cancer, including anogenital and oropharyngeal cancers such as anal, vulvar, vaginal, CC, and penile cancers [1]. Out of the 170 known types of HPV at least 12 are categorized as high risk [2]. Human papillomavirus (HPV) is recognized as the most prevalent sexually transmitted infection (STI) accounting for more than half of all new and existing STIs [3]. The International Agency for Research on Cancer (IARC) has identified 12 strains of HPV that are considered carcinogenic to humans. These contain HPV 16/18/31/33/35/39/45/51/52 /56 /58, and 59 [4]. When the mucosal epithelium is infected with HPV, it can become carcinogenic due to the inhibition of p53 and pRB tumor suppressors by E6 and E7 oncoproteins [5]. High-risk strains of HPV are probable to cause cancer than others, but it may take a long time for the infection to progress to that stage. Other types of HPV are less dangerous and mostly affect the skin and mucosa, causing conditions like warts, abnormal growths, or benign tumors. The outcome of HPV infection relies on various components of the environment, the host, and the virus itself. People with weakened immune systems have a higher chance of getting infected than healthy people [6]. The association between HPV and benign growths like genital warts has been shown [3]. Many HPV infections do not result in any sign and are typically eliminated within a period of two years usually without the need for any medical intervention [7]. Moreover, empirical data suggests that HPV infections are responsible for the majority of ailments leading to both cervical cancers as well a diverse array of malignancies pertaining genitourinary tract and throat region [8].

Persistent cervical infections by high-risk strains are associated with nearly all cases of cervical cancer and the development of its immediate precursor lesions [9]. HR-HPV16/18 are responsible for causing approximately 50% and 20% of cervical cancers, respectively [10]. Cervical cancer emerges as ranked fourth most frequently diagnosed cancer impacting women globally it attains the same ranking in terms of loss of human lives. In addition, it is displayed that close to 604,000 incidents and 342,000 deaths of cervical cancer surfacing globally over the course of 2020 [1, 11].

In 2019 there was an unprecedented number of introductions primarily in low- and middle-income countries (LMIC) that have faced limited access to HPV vaccines. These countries achieved an average coverage rate of approximately 67% for the initial dose and 53% for the final dose. Interestingly. LMICs outperformed high-income countries in terms of initial dose coverage but fell behind when it came to the final dose due to higher dropout rates. It is important to note that only 5 (6%) countries were successful in achieving a final dose coverage rate exceeding 90%. Meanwhile, 22 countries (21%) managed to reach coverage rates of 75% or higher, On the contrary, a staggering 35 (40%) countries had a final dose coverage rate of 50% or less. When we take into account the world population size, it is roughly calculated that universal coverage for the last HPV dose in 2019 was merely around 15% [12]. Research has demonstrated that Gardasil-9 prevents cervical cancer caused by HPV. It has a 92% effectiveness rate in Africa and North America, 91% in Europe, 90% in the Americas Latin and Caribbean, 88% in Asia, and 87% in Australia [13].

DNA vaccines are a new approach to developing HPV vaccines, introducing specific antigens into the vaccinated individual’s cells. These vaccines induce strong, long-lasting immune responses, are easy and cost-effective to produce, and can express multiple antigens simultaneously. However, further research is needed to optimize delivery methods and enhance immunogenicity [14] also Virus-like particle (VLP) vaccines, which mimic the virus’s structure but lack genetic material, have been successful in developing HPV vaccines. These non-infectious particles trigger strong immune responses without infection, effectively preventing HPV infection and associated diseases like cervical cancer [15].

Since 2009, HPV vaccines have been available following the recommendation of the World Health Organization [16]. The main population to receive the vaccination were girls aged 9–14 who would get 2 doses before they became sexually active and girls aged > 15 who would get 3 doses regardless of their HIV status or immune system. Three vaccines that protect against HPV that causes disease are available: quadrivalent (Gardasil), which covers 4 HPV genotypes 6/11/16/18; bivalent (Cervarix), which protects against HPV-16 and HPV-18; and non-avalent (Gardasil-9), which prevents HPV-6/11/16/18/31/33/45/52/58 [17].

A lack of adequate screening programs is the main cause of the difficulties faced by nations that are still in the process of economic development [18]. Cervical cancer is not considered one of the top 10 cancers that affect females in Iran, but it is estimated that it has a yearly incidence of about 947 cases [19].

Several molecular HPV tests have been developed and validated to meet the international standards required for their use in screening programs, for instance, Molecular Pap smear. These tests serve as valuable biomarkers in detecting HPV-related diseases [20].

Given the significance of HPV prevalence and high-risk strains, our goal was to measure the frequency of HPV infection and the associated genotypes that have high or low risk. This study analyzed several individuals to identify whether they were infected with HPV or not and if so, what factors may have contributed to the infection in HPV-positive individuals.

## Methods and materials

### Individuals of the study

We confirm that all methods were carried out in accordance with relevant guidelines and regulations, and that all experimental protocols were approved by the Institutional Review Board (IRB) of the Islamic Azad of Tehran University of Medical Sciences. Informed consent was obtained from all subjects and/or their legal guardian(s).

In this research data from individuals were collected that only were suspected to HPV and no other co-infection was observed.

### Study population

The study conducted a cross-sectional analysis of 936 samples, of which 255 (27.2%) were males and 681 (72.8%) were females. Samples were obtained from oral, vaginal, and genital sites of individuals across six hospitals in Tehran between January 2021 and January 2023. Various medical professionals, including gynecologists, urologists, and dermatologists, collected the samples for the study. People who agreed to participate in the research were asked to fill out a written consent form.

### Sample preparation

For females, swab samples were collected from the vagina and placed in a vial pre-filled with 6 mL of liquid-based cytology (thinprep) at 2–8 °C to prepare for analysis. For male participants, two separate swabs were taken from each genital organ, one from the meatus and the other from the genital area, scrotum, and perianal area. These swabs were stored in a frozen container. In addition, males were asked to gather their first-morning urine in a collection tube, and another urine sample was taken separately. Following collection, the samples were stored at a temperature of -20 °C until the time of analysis. The study’s inclusion criteria comprised females who showed abnormal cytological findings and were recommended by their physicians to undergo HPV detection and typing and males who were suspected to have HPV infection referred to the laboratory.

### DNA extraction and PCR

The pre-amplification procedures, DNA extraction, and HPV genotyping were carried out. The QIAamp DNA extraction kit (Qiagen, Hilden, Germany) was used to extract HPV DNA, based on its structural design. Subsequently, PCR was conducted using the REALQUALITY RQ-Multi HPV detection and genotyping kit (AB-Analitica, Italy) on the extracted genome.

### Statistical analysis

Statistical analysis was done using SPSS software version 27. Frequency was used to describe data and determine HPV prevalence and genotype distribution. Fisher’s exact and chi-square tests were used to compare the prevalence and examine the distribution of genotypes in subgroups.

## Results

The demographic data comprised 936 individuals, out of which 255 (27.2%) were males and 681 (72.8%) were females. These samples were collected from Laboraotories in Tehran between January 2021 and January 2023, to examine the prevalence of HPV. Of the 936 samples, 324 (34.6%) were positive for HPV, while 612 (65.4%) were negative. Among our participants, 70 (27.5%) of males and 254 (37.3%) of females were HPV-positive (Table 1).

### Distribution of HPV genotypes

In this study, 440 HPV genotypes were ascertained in 324 positive cases, which included people who were infected with multiple genotypes. Among the 88 genotypes present in males, a higher proportion of high-risk (14.5%) genotypes were observed compared to low-risk (5.4%) genotypes, indicating that the frequency of high-risk genotypes was higher. A total of 352 genotypes were identified in females, unlike males, had more low-risk (42%) genotypes than high-risk (37.9%) genotypes (Chi-Square: 18.047, p-value < 0.001) (Table 1).

In non-vaccinated people, 391 genotypes were identified, of which 209 (53.4%) low-risk genotypes were more than 182 (46.6%) high-risk genotypes. In non-vaccinated female individuals, 185 (47.3%) were LR and 138 (35.3%) were HR. However, in non-vaccinated male individuals, 24 (6.1%) were LR and 44 (11.3%) were HR (chi-squared: 10.909, p-value < 0.001). But in vaccinated people, 49 genotypes were identified, all of which were high-risk, and low-risk genotypes were not identified. Also, there were no reports of frequent high-risk genotypes such as HPV-16 and HPV-18 in these individuals (Table 2).

**Table 1:** Demographic data and HPV prevalence

Demographic Data	Gender	
	Male	Female
Abundance	255 (27.2%)	681 (72.8%)
Positive	70 (27.5%)	254 (37.3%)
Total Positive	324 (34.6%)	
Total Negative	612 (65.4%)	
Total	936	

Among 324 HPV-positive people of both sexes, the highest to the lowest genotypes included HPV-6, 16, 11, 18, 45, 52, 31, 59, and 56, respectively.

In males, high-risk (HR) genotypes constituted a higher percentage of identified genotypes, which included HPV-16, 18, 52, 56, 31, 45, and 59, respectively, from the highest to the lowest, while low-risk (LR) genotypes 6 and 11 had an equal amount.

Unlike males, more low-risk genotypes than high-risk genotypes were identified in females, which included HPV-6, 16, 11, 18, 45, 31, 52, and 59, respectively, from the highest to the lowest.

Although high-risk genotype 18 (13.6% prevalence in both sexes) was detected more frequently in females, it had a higher prevalence in males. While the number of people with high-risk genotype 16 (35.2% prevalence in both sexes) was very different between the sexes, the prevalence of this genotype was close to the same in both sexes. Low-risk genotype 6 (35.2% prevalence in both sexes) was the most common in females but the third most common genotype in males (Table 2).

There were differences between males and females in terms of the high-risk genotypes that had the lowest prevalence. For instance, genotype 56 had a low prevalence in males (8.6%) and was not observed in females. In addition, genotype 52 had a prevalence of 5.6% in both sexes. The high-risk genotypes 59 (3.7%) and 56 (1.9%) were identified as the least common in both sexes. The remaining genotypes were present at varying percentages and with low prevalence among both females and males (Table 2).

In addition, this study identified several differences in the prevalence and distribution of HPV genotypes between males and females. For example, the prevalence of different high-risk genotypes was higher in males than in females.

**Table 2:** Distribution of HPV positive genotypes by gender

Distribution of HPV Positive Genotypes	Gender	
	Male	Female
High Risk Genotypes in non- Vaccinated People	16, 18, 52, 56, 31, 45, 59	16, 18, 45, 52, 31, 52, 59
	44 ( 62.8%)	96 ( 37.9%)
Low Risk Genotypes in non- Vaccinated People	6, 11	6, 11
	24 ( 34.2%)	106 ( 42%)
Genotypes in Vaccinated People	2 (2.85%)	29 (20.4%)
Total High Risk Genotypes in non- Vaccinated People	209 ( 53.4%)	
Total Low Risk Genotypes in non- Vaccinated People	182 (46.6%)	
Total Positive Genotypes	88 (20%)	352 (80%)
	440	

## Discussion

HPV is classified as the most frequent STI globally which has on its record HR-HPVs causing infiltrative lesions in genital and oropharyngeal regions whilst LR-HPVs correlate with larger development risk for Condyloma symptoms manifestation(s). In light of our latest study findings indicating that HPV-16 had an outsized share among males and females infected making it incessantly pervasive amongst genotypes. Another important finding is more cases were reported among men for HPV-18 and that there had been a sizeable incidence of LR-HPV 6/11. Yet, people can take advantage of vaccine coverage that deals with these strains. It is fundamental to note that factors such as inadequate knowledge, absence of awareness about transmission routes, and skepticism about the efficacy of vaccination methods all contribute to higher prevalence rates.

Although all current HPV vaccines protect HPV-16, this high-risk genotype was still the most commonly reported HPV in unvaccinated groups of our study which diminish the frequency of HR-HPV genotypes and control the prevalence. According to a study by Hassani, et al.; genotype 16 was the most frequent among 2562 females aged 15–59 years from the general public residing in 11 provinces of Iran. This outcome was consistent for both sexes [19]. Similar outputs have been announced in 2022 by Shalchimanesh, et al.; on 219 people (160 females and 59 males) with HPV infection from Tehran, Iran [21]. A few percent of vaccinated individuals were infected with either genotype under vaccine coverage or some lesser-known genotypes due to high confidence in complete vaccine immunity. To emphasize the vaccination trend a study in Stockholm demonstrated the percentage of females who received the vaccine rose from 10.7% (2008–2010) to 82.1% (2017–2018). Also, in comparison to 2008–2010 (34.7%), the results of HPV-11, 16, 18 prevalence in non-vaccinated females were higher than in 2017–2018 (16.7%) [13]. It is notable from our research results that unvaccinated people have frequently reported possessing specific genotypes compared to those who have been vaccinated. The study indicates how important vaccination can be in reducing exposure and preventing high-risk genotypes, which are linked with cancer development. A similar outcome has been observed in aligned research by Kamolratanakul, et al.; that indicates HPV vaccination has strong effectiveness against oral HPV-18, and HPV-16, and also prevents cancers associated with genotypes 16, 18, 6, and 11 [17].

Fluctuations and differences in the abundance of genotypes vary due to geographic conditions. Despite our study that showed the most involved females were infected by 6, and 16 genotypes (55%) a study by Olia, et al.; in Urmia on suspected females indicated that the most infected individuals (53.4%) were associated with low-risk genotypes (6, 11, 26, 53, 67) [22]. By contrast, research by Mboumba Bouassa, et al.; performed on 253 females suspected of HPV, discovered that the percentage of HR-HPVs among the infected was as considerable as 68.9 which were 31-52-56-35-58-16-45-52 genotypes. Only 58 − 35 genotypes were not seen in our examination [23]. In another analysis by Ährlund-Richter, et al.; at a Youth clinic in Stockholm from females aged 15 to 23, 178 cervical swabs were collected and analyzed and the data on vaccination status gathered and found that the most frequent genotypes in both vaccinated and non-vaccinated females were 39, 51, 52, 56, and 59 [13].

Based on our findings it appears that receiving the HPV vaccination does not eliminate individual susceptibility to uncommon high-risk HPV genotypes like 59, 52, and 56 which pose a high risk for infection. Our research suggests this might result from an insufficient understanding of the safety profile and effectiveness of vaccines fueled by misplaced confidence in their immunization status, and risky sexual behavior. It is essential to recognize that engaging in high-risk sexual behaviors heightens the probability of catching HPV infections. Our research collaborates closely with the findings made by Ghobadi, et al.; which identified various risk factors related to HPV spread. Some prevalent traits include commencing sexual activity prematurely, indulging in frequent sex acts, and partaking in multiple partner sexual experiences while overlooking genital cleanliness; additionally having intimate relations with men who participate in unprotected sex activities poses a significant danger [24]. According to the findings of Cossellu, et al.; studies on HPV, the risk factor was similar [3]. Moreover, STIs can spread via sexual contact, such as vaginal, oral, and anal sex, for instance, HIV, syphilis, gonorrhea, and genital herpes [25]. They create a strong background for co-infected diseases like HPV by suppressing the immune system. A study from 145 infected individuals among 292, subjects documented the prevalence of HPV is higher (51%) among the STIs group than the healthy population(43%) [26]. The study also showed that insufficient information is a key factor for the non-uptake of vaccination. It is essential to provide active education about how HPV infection develops and how vaccination can help to improve vaccination rates throughout the country [27]. In a study by Osazuwa Peters, et al.; 63% of the 301 participants who completed the questionnaire were men. Nearly 45% and 71% of partakers reported having five or more oral and vaginal sex partners, respectively. Men had more oral and vaginal sex partners than females, started both at a younger age and had less education. The study emphasizes that the foremost element that boosts the incidence of getting infected and having persistent HPV is engaging in unsafe and unprotected sexual activities [28]. A study on human papillomavirus prevalence in unvaccinated heterosexuals by Menby Machalek, et al.; among 511 unvaccinated males demonstrated, LR-HPV 6/11 had a prevalence of 1.1% and 1.5% in men aged 16–20 or 21–25 years, respectively, and this remarkably jumped to 9.3% and 6.1% in males aged 26–30 or 31–35 years, respectively. Similarly, high-risk HR-HPV 16/18 had a prevalence of 1.6% and 2.2% in men aged 16–20 and 21–25 years, respectively, and this increased notably to 9.3% and 4.9% in men aged 26–30 and 31–35 years, respectively. By and large, age is a factor that strongly influences the prevalence of HPV genotype [29].

Even though we adequately studied the subject and reached acceptable results further studies with larger study groups and more variables are required to properly tackle this issue. By increasing the statistical population, we can reach more accurate results on HPV prevalence. The outcomes obtained from our data can be generalized to populations in developing countries where vaccination programs need to be more precise.

In conclusion, the findings of this research indicate a considerable prevalence of HPV infection in both males and females. For example, the prevalence of high-risk genotypes such as genotype 18 was reported in males more than in females. As it turned out, genotypes 16 and 18 were more common in males, while among both genders genotype 6 emerged as the most prevalent genotype in females, and simultaneously ranking as the third most common genotype in males. Genotype 11 was among the four most common genotypes in both groups and high-risk genotype 56 was present only in males with a low prevalence. Other genotypes had different prevalences in males and females. Also, the most common high-risk HPV genotypes in both sexes were 16, 18, 45, 52, 31, and 59, respectively, while the most common low-risk genotypes were 6 and 11. This study also emphasizes the importance of HPV vaccination in decreasing HPV infection, as it has been confirmed to be an effective method in reducing the prevalence of genotypes that are covered by vaccination, but these individuals may be exposed to other strains that are not covered. These findings highlighted the need for targeted education programs to promote HPV vaccination and safer sex practices, particularly among younger populations who may be at risk for high-risk HPV genotypes.

## Data Availability

The raw data required to reproduce these findings are available from the corresponding author upon request.
